# Radiomics Combined with Transcriptomics Improves Prediction of Breast Cancer Recurrence, Molecular Subtype and Grade

**DOI:** 10.3390/cancers17172912

**Published:** 2025-09-05

**Authors:** George K. Acquaah-Mensah, Boris Aguilar, Kawther Abdilleh

**Affiliations:** 1 Department of Pharmaceutical Sciences, School of Pharmacy-Worcester/Manchester, Massachusetts College of Pharmacy and Health Sciences , Worcester, MA 01608, USA; 2 Institute for Systems Biology, Seattle, WA 98109, USA; baguilar@systemsbiology.org; 3 Pancreatic Cancer Action Network, El Segundo ,CA 90245, USA; kabdilleh@pancan.org

**Keywords:** radiomics, breast cancer, machine learning, disease recurrence, molecular subtype

## Abstract

**Background/Objectives:** Breast cancer (BrCA) is among the deadliest cancers for women in the world. The disease has four distinct molecular subtypes which can be determined by gene expression profiling. Understanding these subtypes has enabled the development of targeted therapeutics. Additionally, following initial successful treatment, some patients experience disease recurrence events. **Methods:** In this study, we used radiomics coupled with machine learning techniques to predict molecular subtypes and disease recurrence events from a dataset of MRI features deriving from a single-institutional, retrospective collection of 922 biopsy-confirmed invasive BrCA patients. The feature-rich and comprehensive dataset consists of radiomic as well as demographic, clinical, and molecular subtype information. We focused our analyses on Black and White patients who were 50 years or younger at diagnosis (n = 346) to identify racial disparities that exist between molecular subtypes and disease recurrence events. Random Forest and AdaBoostM1 were applied to over 500 radiomics features. **Results:** Radiomics alone or combined with gene expression data can accurately predict molecular subtype and disease recurrence events for both racial groups. In total, we found over 40 radiomics features that have significant associations with race. The radiomic features that are most predictive in the Breast and Fibroglandular Tissue Volume imaging category for Black patients was breast volume (Breast_Vol) and for White patients was post contrast tissue volume (TissueVol_PostCon). **Conclusions:** These results suggest that radiomics can be used to predict differences in BrCA recurrence and molecular subtype between racial groups and can have an impact on clinical outcomes.

## Simple Summary

Breast cancer (BrCA) is among the deadliest cancers for women in the world. There are different presentations of BrCA that can be explained by molecular delineations called molecular subtypes. Molecular subtypes are yet to be fully characterized. Moreover, some patients experience disease recurrence after successful treatment. Clinical radiology imaging data such as from MRIs are a rich resource that can be interrogated to help research in such studies. Using disparate data types, including imaging, clinical and gene expression data, we characterized the differences between Black and White BrCA patients in terms of subtypes and disease recurrence using machine learning approaches. We found that combining radiology imaging and molecular data improved machine learning predictions of subtypes and disease recurrence for both racial groups. Accurately predicting subtypes and recurrence between the racial groups can have an impact on clinical outcomes and allow for more precise tailoring of treatments to patients.

## 1. Introduction

Breast cancer is among the deadliest cancers for women in the world [1]. A large body of research has been carried out to understand and characterize breast cancer at the molecular level, resulting in the molecular classification of breast cancer subtypes [2,3,4]. To date, breast cancer has been found to have at least four distinct molecular subtypes, which can be determined by gene expression profiling: Luminal A (Hormone Receptor (HR)-positive and Human Epidermal Growth Factor receptor 2 (HER2)-negative), Luminal B (HR+/HER2+), Triple-Negative Breast Cancer (TNBC), and HER2+ [5]. Racial disparities exist among breast cancer patients across the various molecular subtypes. For example, Black breast cancer patients are more likely to present with more aggressive disease compared to White breast cancer patients. In addition to disparities in socioeconomic barriers between the groups, differences in the biological expression of disease are also observed. Understanding the different breast cancer subtypes has enabled the development of targeted therapeutics. Further exploration and deeper understanding will facilitate early detection strategies and improve patient outcomes [6].

A recent study comparing White and Black breast cancer patients across a large University of Chicago Comprehensive Cancer Center cohort found that racial disparity existed between White and Black breast cancer patients in terms of both survival and recurrence, and a racial disparity within the Luminal A subtype in both overall survival and recurrence-free survival [7].

After initial successful treatment, breast cancer re-occurs in some cases, arising out of cells from the original tumor. Therapeutic and surgical interventions are helpful in reducing breast cancer recurrence rates. For instance, five years of adjuvant endocrine treatment decreases the risk of disease recurrence in estrogen receptor-positive (ER+) breast cancer substantially [8,9]. Breast-conserving surgeries have been reported to result in an 8-year low locoregional recurrence rate of 3.2% [10]. Moreover, in a Danish population, the use of HMG-CoA reductase inhibitor drugs has been reported to be associated with reductions in 5-year breast cancer recurrence rates [11]. Disease recurrence is observed in certain patients but not in others. Factors responsible for disease recurrence remain a subject of intense research interest [12,13]. TNBC patients have a higher propensity for disease recurrence [14,15]. Some 30–40% of TNBC patients experience recurrence within 5 years, in operable settings [16]. Recurrence rates are even higher among patients who do not achieve initial pathologic complete response (pCR), although patients with the more aggressive TNBC or those who are HER2-positive are more likely to achieve pCR [17,18].

Radiomics is an approach that combines computer science, medical imaging, and artificial intelligence to extract multiple relevant features from digital medical images. Recently, radiomics has been successfully used to differentiate between molecular subtypes [19,20,21], predict disease recurrence [22], and characterize differences in MRI features between racial/ethnic groups [23].

Here, using machine learning methods, we combined radiomics, clinical, and gene expression data with race data to identify disparities in MRI features between Black and White breast cancer patients. We identified MRI features that had a significant association with race, including features used to distinguish between benign and malignant tissue. We also identified differences between the most predictive features associated with disease recurrence. We demonstrated the utility of radiomic features in predicting disease recurrence events, molecular subtype, and Nottingham grade. All of these are potential facilitators for precision medicine.

## 2. Materials and Methods

A workflow scheme of our methodology is depicted in Figure S10 of the Supplementary Material.

### 2.1. Patient Cohort Characteristics

The original cohort from Saha et al. [24] (922 patients) was filtered down to include White or Black non-Hispanic patients who were 50 years or younger (n = 347; 270 White patients and 77 Black patients). Detailed patient cohort characteristics can be found in Table 1. The breakdown of molecular subtype information where available before class sizes included the following: Luminal-like (0) (n = 226); [Estrogen Receptor- or Progesterone Receptor-positive (ER/PR+) and HER2+] (1) (n = 36); HER2+ (2) (n = 20); Triple-negative (TN) (3) (n = 35). In total, 29 patients (22 White and 7 Black) had recurrence events, while 318 patients did not (248 White and 70 Black).

### 2.2. Data Source

Data used in this study derives from Saha et al. [24]. It includes dynamic contrast-enhanced MRI images from a single-institutional, retrospective collection of 922 preoperative, biopsy-confirmed invasive breast cancer patients collected over a decade. The dataset consists of 529 MRI derived features as well as detailed demographic, clinical, pathology, treatment, outcomes, and molecular subtype information. Table S2 (Supplementary Material) lists the names of the radiomic features with descriptions and names of the corresponding radiomic categories.

### 2.3. Transcriptomic Data from TCGA

The Institute for Systems Biology-Cancer Gateway in the Cloud (ISB-CGC) hosts the TCGA data in Google BigQuery tables (www.isb-cgc.org). Using annotations from the Gene Ontology [25], genes that were either oncogenes or tumor suppressors were selected from the TCGA BRCA RNASeq data.

### 2.4. Machine Learning

#### 2.4.1. Machine Learning Workbench

The Waikato Environment for Knowledge Analysis (WeKa, version 3.9.6) workbench, which has Java implementations of a variety of machine learning schemes, was used for machine learning as specified below [26].

#### 2.4.2. Balance Class Sizes—SMOTE

To enhance performance, the Synthetic Minority Over-sampling Technique (SMOTE) was used to balance class sizes [27]. Under this, synthetic instances were generated for the minority class, using Nearest Neighbors. The number of Nearest Neighbors used was set at the default value of five. Where there were more than two classes involved, the implementation in the R CRAN package (R version 4.3.2), smotefamily (version 1.4.0), was used [28]. For two-class balancing, the WeKa SMOTE implementation was used. After class sizes were balanced for molecular subtype, the breakdown was as follows: Luminal-like (0), n = 226; [ER/PR+, HER2+] (1), (n = 262); HER2+ (2), (n = 200); TN (3), (n = 260). After class sizes were balanced for recurrence events for all subjects, the breakdown was as follows: no (n = 318), yes (n = 319). After balancing class sizes for recurrence events in White patients, the breakdown was as follows: no (n = 248), yes (n = 242); in Black patients, the breakdown was as follows: no (n = 70), yes (n = 70).

#### 2.4.3. Decision Tree Splits and Information Gain

The J48 decision tree builder is a variant of the C4.5 decision tree-building method [29,30]. Thus, the J48 method uses Information theory. Specifically, it builds decision trees based on attribute values of already classified instances in the training dataset. For a classification task, the data are divided based on attribute range values found in the training set. The features with the greatest normalized information gain are the ones upon which decision splits are based. We implemented J48 and used default parameters in WeKa.

#### 2.4.4. Feature Selection

The Boruta algorithm was used to select the most important attributes in the training data [31]; Boruta uses a Random Forest algorithm to compute Importance Scores for each attribute relative to a background of randomly generated attributes (values for which are randomly generated from shuffled values from training set attribute values). An R implementation of Boruta (version 9.0.0) was used [31]. The number of runs used was 1000. All other parameters were default parameters.

#### 2.4.5. Correlation Analysis

The imaging features and clinical tables from Saha et al. [24] were transformed into Google BigQuery [32] tables to perform statistical analysis. Wilcoxon sum-rank correlations were computed between the imaging features and race and other clinical features.

## 3. Results

### 3.1. Prediction of Molecular Subtypes

Three machine learning algorithms were trained with radiomics data alone, gene expression data alone, or a combination of the two. Table 2 summarizes and compares the performance of algorithms based on F-Measures. Overall accuracy for the same models is reported in Table S4 of the Supplementary Material. Gene expression data consisted of expression values for genes annotated as oncogenes or tumor-suppressor genes in the Gene Ontology. The machine learning schemes were the J48 Decision Tree algorithm, the Sequential Minimal Optimization (SMO) support vector machine, and a Multi-Layer Perceptron. With the exception of molecular subtype 0 (luminal-like), class prediction improved when the training class sizes were balanced. For example, SMO 10-fold cross-validation F-measure values for molecular subtypes 0, 1, 2, and 3 were 0.776, 0.164, 0.061, and 0.278, respectively, when class sizes were not balanced and radiomics data alone were used. After balancing class sizes, the corresponding values were 0.776, 0.951, 0.971, and 0.899, respectively.

Similarly, using the J48 and 10-fold cross-validation for balanced class size, F-measure values were 0.676, 0.86, 0.976, and 0.889, respectively, for molecular subtypes 0, 1, 2, and 3 when radiomics and gene expression data were used. This is an improvement on when gene expression data alone were used (corresponding F-measure values: 0.635, 0.809, 0.976, and 0.871) or radiomics data alone were used (corresponding F-measure values: 0.634, 0.843, 0.859, and 0.766) (Table 2).

### 3.2. Prediction of Recurrence Events

To determine if there were radiomic features that were better predictors of disease recurrence events in one group over the other, we conducted machine learning experiments using the tree-based ensemble learning schemes: Random Forest and AdaboostM1 (using Random Forest). The algorithms were trained with different categories of radiomics data, in accordance with the categorization used by Saha et al. (2017) [24]. Regarding disease recurrence events, there were two classes to be predicted: “yes” or “no”. Ten-fold cross-validation was used in each instance.

**Figure 1:**
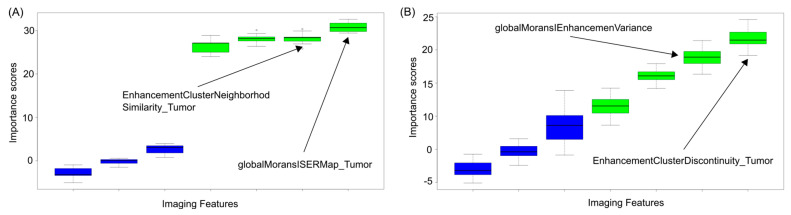
(**A**,**B**)**.** The highest-scoring predictive MRI features for disease recurrence differ between Black and White patients. As an example, (**A**) the most predictive Tumor Enhancement Spatial Heterogeneity (TESH) feature for disease recurrence in Black patients 50 years old or younger is EnhancementClusterDiscontinuity_Tumor (Mean Importance Score as computed using the Boruta algorithm: 16.7). In contrast, the most predictive TESH feature for disease recurrence similarly computed for White patients 50 years old or younger is (**B**) globalMoransISERMap_Tumor (Mean Importance Score: 30.8). Blue box plots represent background attributes randomly generated from shuffled values. Green box plots represent original attributes and have Importance Scores higher than those of the background (blue) ones.

**Figure 2:**
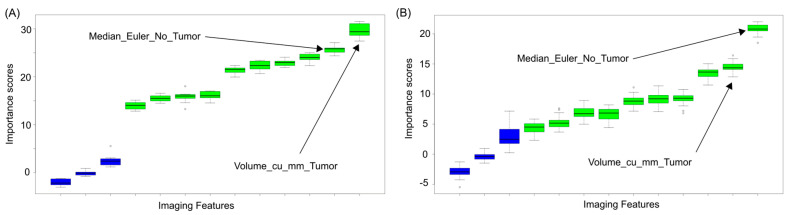
(**A**,**B**). In other instances where the highest-scoring predictive feature is the same for both groups, the Importance Scores computed for that feature differ between the groups. As an example, (**A**) Tthe most predictive Tumor Size and Morphology (TSM) feature for disease recurrence in Black patients 50 years old or younger is Median_Euler_No_Tumor (Mean Importance Score as computed using the Boruta algorithm: 20.8). Similarly, the most predictive TSM feature for disease recurrence computed for White patients is (**B**) Median_Euler_No_Tumor, but the Importance Score computed is higher (Mean Importance Score: 29.7). Blue box plots represent background attributes randomly generated from shuffled values. Green box plots represent original attributes and have Importance Scores higher than those of the background (blue) ones.

We identified the most predictive features of disease recurrence in the different categories, and in most cases, they differed between White and Black patients (Figure 1 and Figure 2). Figure 1 shows that the order in Importance Scores (refer to Section 2.4.4 for how Importance Scores were determined) changes between Black and White patients considering the Tumor Enhancement Spatial Heterogeneity (TESH) (Figure 1A,B). On the other hand, the order is the same in Black and White patients considering the Tumor Size and Morphology (TSM) category, but the magnitude of Importance Scores differs between the two groups (Figure 2A,B).

The algorithms’ prediction performance (F-measure) was better with Black patients than with White patients with the following feature categories: Breast and Fibroglandular Tissue (FGT) Volume Features, Tumor and FGT Enhancement, FGT Enhancement, Enhancement Texture, FGT Enhancement Variation, Tumor Enhancement, and Tumor Enhancement Texture. In contrast, the algorithms’ prediction performance when trained with Tumor Enhancement Spatial Heterogeneity, Tumor Enhancement Variation, or Tumor Size and Morphology (10 features within this category) categories was better in White patients than Black patients based on the F-Measures reported in Table 3 and Supplementary Table S1. Additional performance metrics for predicting recurrence events for Black and White participants are reported in Table S5 of the Supplementary Material.

### 3.3. Prediction of Nottingham Grade

Table 4 summarizes prediction performance (F-Measure) of the J48, Random Forest (by itself), and AdaboostM1 (using Random Forest) methods using imaging data alone or imaging data with some clinical data, including menopausal status at diagnosis, race and ethnicity, metastatic status, and ER/PR/HER2 status. The classes to be predicted were Nottingham Grades 1, 2, or 3. Additional metrics of prediction performance are included in Table S6 of the Supplementary Material. Without exception, performance improved when class sizes in the training data were balanced. Without exception, performance improved when imaging data were combined with clinical data than when only imaging data were used.

### 3.4. Association Analysis Between Radiomic/Clinical Features and Race

#### 3.4.1. Association Analysis Between Clinical Features and Race

Using Google BigQuery, we identified clinical features that were significantly associated with race using the Chi-Squared test between race and categorical clinical features. There is a significant difference between Black and White patients in the features that are listed in Table 5. Most significant features associated with race include Lymphadenopathy_or_Suspicious_Nodes, Estrogen Receptor status (ER), Molecular Subtype, Tumor Grade Mitotic and Nuclear, and Progesterone Receptor status (PR).

#### 3.4.2. Association Analysis Between Radiomic Features and Race

Using Google BigQuery, we identified radiomic features that were significantly associated with race. There is a significant difference between Black and White patients in the features that are listed in Table 6. Most significant features associated with race include those used to distinguish between benign and malignant tissue, more specifically, those relevant to signal enhancement ratio (SER) and washout pattern (Table 6) [33].

## 4. Discussion

Accurate predictions of molecular subtypes, disease recurrence, and Nottingham grade are critical for better diagnosis, treatment, disease management, and surveillance of breast cancer patients [34,35,36]. Moreover, there is a poorer prognosis in Black women compared to White women 50 years of age or younger and at stage II. The molecular underpinnings that govern this difference remain unclear. In this work, we trained a set of machine learning models to predict these three endpoints from features derived from medical images as well as gene expression data. To predict differences between Black and White breast cancer patients, we identified image-derived features that are significantly associated with race.

In Table 6, we list image-derived features that are significantly associated with race, including those features that help to distinguish between benign and cancerous tissue. One of those features is known as signal enhancement ratio (SER). Certain dynamic contrast-enhanced breast MRI (DCE-MRI) parameters, including parameters related to SER, correlate with micro-vessel density, which is a surrogate for angiogenesis [37], an important cancer hallmark. Furthermore, it was reported that high values of SER are an independent predictor for disease recurrence in triple-negative breast cancer patients [38]. In addition, early-phase MRI SERs are predictive of pathological complete response following neoadjuvant chemotherapy [39].

This work also highlighted several MRI-derived features that are related to relative signal intensity called wash-in rate (WIR). It has been shown that WIR is an acceptable diagnostic parameter for borderline/malignant lesions in female reproductive organs, including the uterus and ovaries [40]. Likewise, WIR is an important parameter that helps distinguish between prostate cancer lesions and non-cancer lesions as well in testicular tumors [41,42]. Our results show that parameters related to WIR also help distinguish between Black and White breast cancer tumors.

We surveyed available radiomic features, including SER features extracted from breast cancer patients in the TCGA BRCA project, as a validation dataset. However, we were unable to test for differences between the groups because of the limited number of Black patients for whom both radiomics and molecular data were available. More multi-modal datasets from diverse populations will be crucial to furthering our understanding of the biological differences between groups, allowing for more tailored therapeutics.

**Figure 3:**
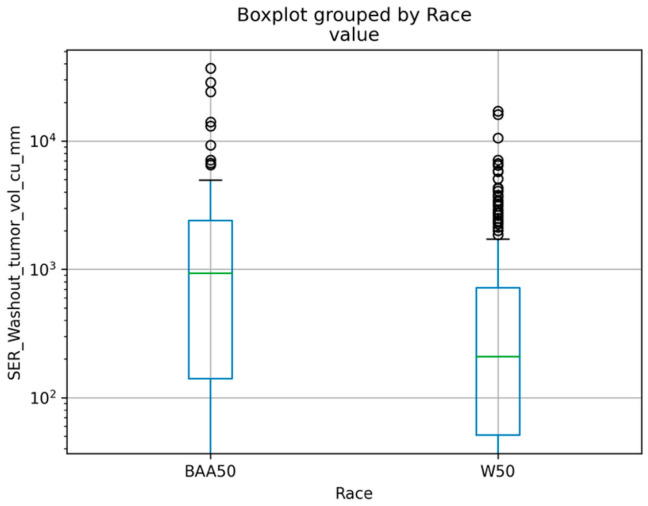
The MRI feature, SER_Washout_tumor_vol_cu_mm, is significantly associated with race (*p* < 0.0008). BAA50 (Black or African American 50 years or younger); W50 (White 50 years or younger). The MRI features described in Saha et al. (2017) [24] supplement B, and the corresponding clinical tables were transformed into Google BigQuery tables where Wilcoxon sum-rank correlations were computed between the imaging features and race and other clinical features.

This study is unique in that it combines radiomics, clinical, and gene expression data to identify differences between Black and White breast cancer patients. We identified more than 30 MRI features that had a significant association with race (*p*-adj < 0.01) (Figure 3, Supplementary Figures S1–S9, and Table 5). We predicted disease recurrence events, molecular subtype, and Nottingham grade using machine learning algorithms, and we obtained good performance using Random Forest and J48 Decision Tree. The incidence of higher-grade disease and more advanced stage disease can be associated with a Black population due to access to diagnosis or other socioeconomic [43] factors. However, according to the Chi-Squared test (Table S3), the association between race and grade in the dataset used in this work (Section 2.2) is not significant (*p* > 0.05). Additional data and analysis are required to include socioeconomical factors in the analysis.

For disease recurrence, we identified the most important radiomic features that would predict outcomes depending on race. For molecular subtype, the performance of machine learning was better with a combination of gene expression and radiomic features than with either data type alone. In the case of Nottingham grade, performance improved when radiomic data were combined with clinical data than when only radiomic data were used.

## 5. Conclusions

Using disparate data types, including radiomics, clinical, and gene expression data, we characterized the differences between Black and White breast cancer patients in terms of disease recurrence and molecular subtype. We used machine learning algorithms to predict disease recurrence as an outcome and identified MRI features that are both predictive of disease recurrence and differ between the groups. Moreover, we found that combining radiomic and molecular data improved machine learning performance as it relates to the prediction of molecular subtype. Accurately predicting molecular subtype allows for more precise tailoring of treatments to patients.

Out of 500 radiomic features investigated, over 40 radiomics features had significant associations with race. Radiomics can be used to predict differences in BrCA recurrence and molecular subtype between racial groups.

## Figures and Tables

**Table 1:** Cohort characteristics.

	Black	White	All
Cohort size	77	270	347
Luminal-like	37	189	226
[ER/PR+, HER2+]	8	28	36
HER2+	5	15	20
TN	27	38	35
Recurrence events	7	22	29

**Table 2:** Summary of machine learning performance on classifying subtypes using different data modalities.

		Imaging Data Only		Gene Expression Data Only		Imaging plus Gene Expression Data	
		^$^ Unbalanced Class Size	^^^ Balanced Class Size (After Applying SMOTE)	^&^ Unbalanced Class Size	^##^ Balanced Class Size (After Applying SMOTE)	^&^ Unbalanced Class Size	^##^ Balanced Class Size (After Applying SMOTE)
**Classifier (using 10-fold cross-validation)**	Molecular subtype (Class *)	F-Measure	F-Measure	F-Measure	F-Measure	F-Measure	F-Measure
J48	0	0.682	0.634	0.595	0.635	0.65	0.676
	1	0.205	0.843	0.24	0.809	0.211	0.86
	2	0.083	0.859	0	0.976	0	0.976
	3	0.203	0.766	0.684	0.871	0.529	0.889
SMO	0	0.776	0.776	0.738	0.758	0.747	0.841
	1	0.164	0.951	0	0.884	0.222	0.957
	2	0.061	0.971	0	0.988	0	0.964
	3	0.278	0.899	0.837	0.951	0.667	0.925
Multi-Layer Perceptron	0	0.738	0.449	0.675	0.758	0.771	0.841
	1	0.067	0.536	0.087	0.894	0	0.946
	2	0	0.55	0	0.976	0	0.976
	3	0.198	0.549	0.769	0.951	0.732	0.925

* Class definition: 0 --> luminal-like; 1 --> ER/PR pos, HER2 pos; 2--> HER2; 3 --> Triple-Negative. ^$^ Class size: 0--> n = 226; 1--> n = 36; 2--> n = 20; 3--> n = 35. ^^^ Class size: 0--> n = 226; 1--> n = 262; 2--> n = 200; 3--> n = 260. ^##^ Class size: 0--> n = 38; 1--> n = 44; 2--> n = 40; 3--> n = 40. ^&^ Class size: 0--> n = 38; 1--> n = 11; 2--> n = 5; 3--> n = 20.

**Table 3:** Recurrence events: machine learning performance differences.

		^#^ All Patients		^$^ White Patients		^^^ Black Patients	
	Classifier	Random Forest	AdaBoostM1 (Using Random Forrest)	Random Forest	AdaBoostM1 (Using Random Forrest)	Random Forest	AdaBoostM1 (Using Random Forrest)
**Attribute category**	Recurrence events	F-Measure	F-Measure	F-Measure	F-Measure	F-Measure	F-Measure
Breast and ^&^ FGT Volume Features (n = 5)							
	no	0.728	0.753	0.757	0.751	0.818	0.785
	yes	0.728	0.757	0.758	0.751	0.825	0.8
Combining Tumor and FGT Enhancement (n = 18)							
	no	0.866	0.853	0.889	0.89	0.891	0.891
	yes	0.87	0.861	0.894	0.897	0.895	0.895
FGT Enhancement (n = 82)							
	no	0.898	0.902	0.879	0.874	0.949	0.95
	yes	0.901	0.903	0.884	0.877	0.951	0.95
FGT Enhancement Texture (n = 176)							
	no	0.922	0.922	0.925	0.927	0.964	0.964
	yes	0.927	0.927	0.928	0.93	0.965	0.965
FGT Enhancement Variation (n = 34)							
	no	0.897	0.887	0.903	0.886	0.941	0.933
	yes	0.899	0.887	0.901	0.89	0.944	0.938
Tumor Enhancement (n = 30)							
	no	0.876	0.879	0.895	0.903	0.916	0.948
	yes	0.888	0.888	0.904	0.909	0.926	0.952
Tumor Enhancement Spatial Heterogeneity (n = 4)							
	no	0.858	0.865	0.853	0.859	0.667	0.61
	yes	0.868	0.874	0.853	0.855	0.739	0.719
Tumor Enhancement Texture (n = 135)							
	no	0.923	0.931	0.93	0.921	0.957	0.932
	yes	0.93	0.937	0.931	0.924	0.958	0.939
Tumor Enhancement Variation (n = 35)							
	no	0.94	0.95	0.957	0.956	0.957	0.937
	yes	0.944	0.952	0.958	0.958	0.958	0.934
Tumor Size and Morphology (n = 10)							
	no	0.856	0.863	0.876	0.878	0.826	0.843
	yes	0.873	0.876	0.879	0.881	0.831	0.843

^#^ Ten-fold cross-validation; originally no (n = 318) and yes (n = 29); balanced via synthetic instances generated using SMOTE to no (n = 318) and yes (n = 319). ^$^ Ten-fold cross-validation; originally no (n = 248) and yes (n = 22); balanced via synthetic instances generated using SMOTE to no (n = 248) and yes (n = 242). ^^^ Ten-fold cross-validation; originally no (n = 70) and yes (n = 7); balanced via synthetic instances generated using SMOTE to no (n = 70) and yes (n = 70). ^&^ FGT-> Fibroglandular tissue.

**Table 4:** Prediction performance of J48, Random Forest, and AdaboostM1 (using Random Forest) using imaging data alone or imaging data with some clinical data.

		Imaging Data Only		Imaging and Some Clinical Data	
		^&^ Unbalanced Class Size	^##^ Balanced Class Size (After Applying SMOTE)	^&&^ Unbalanced Class Size	^####^ Balanced Class Size (After Applying SMOTE)
**Classifier (using 10-fold cross-validation)**	**Nottingham grade (Class)**	F-Measure	F-Measure	F-Measure	F-Measure
J48	1	0.22	0.789	0.864	0.969
	2	0.65	0.622	0.953	0.953
	3	0.443	0.78	0.968	0.973
Random Forest	1	0	0.905	0.049	0.917
	2	0.759	0.748	0.761	0.803
	3	0.329	0.884	0.426	0.851
AdaboostM1 (Random Forest)	1	0	0.911	0.049	0.934
	2	0.753	0.73	0.773	0.828
	3	0.345	0.884	0.414	0.87

^&^ Class size: 1--> n = 40; 2--> n = 157; 3--> n = 65. ^##^ Class size: 1--> n = 160; 2--> n = 157; 3--> n = 195. ^&&^ Class size: 1--> n = 40; 2--> n = 157; 3--> n = 65. ^####^ Class size: 1--> n = 160; 2--> n = 157; 3--> n = 130.

**Table 5:** Clinical features significantly (*p*-values < 0.05) associated with race as derived from BigQuery analysis.

Feature	Chi2	Dof	*p*-Value
Lymphadenopathy_or_Suspicious_Nodes	27.8	1	1.35 × 10^−7^
ER	14.8	1	1.17 × 10^−4^
Mol_Subtype	18.6	3	3.28 × 10^−4^
Tumor_Grade_Mitotic	17.5	3	5.54 × 10^−4^
PR	10.4	1	1.28 × 10^−3^
Nottingham_grade	12.5	3	5.84 × 10^−3^
FOV_Computed__Field_of_View__in_cm	31.4	15	7.70 × 10^−3^
Neoadjuvant_Chemotherapy	9.2	2	1.02 × 10^−2^
Tumor_Grade_Nuclear	10.6	3	1.41 × 10^−2^
Clinical_Response__Evaluated_Through_Imaging_	10.6	3	1.44 × 10^−2^
Adjuvant_Endocrine_Therapy_Medications	7.9	2	1.92 × 10^−2^
Overall_Near_complete_Response___Looser_Definition	11.6	4	2.05 × 10^−2^
Received_Neoadjuvant_Therapy_or_Not	7.6	2	2.25 × 10^−2^
Pathologic_response_to_Neoadjuvant_therapy___Pathologic_stage__M__following_neoadjuvant_therapy	9	3	2.96 × 10^−2^
Overall_Near_complete_Response___Stricter_Definition	10.3	4	3.61 × 10^−2^
Pathologic_response_to_Neoadjuvant_therapy___Pathologic_stage__N__following_neoadjuvant_therapy	11.6	5	4.01 × 10^−2^

Chi2—Chi-Squared statistic. Dof—Degrees of freedom.

**Table 6:** Imaging features significantly associated (adjusted *p*-values < 0.05) with race as derived from BigQuery analysis.

Imaging Features	*p*-Adj
SER_Washout_tumor_vol_cu_mm	8.30 × 10^−4^
SER_Partial_tumor_vol_cu_mm	8.38 × 10^−4^
SER_Total_tumor_vol_cu_mm	1.30 × 10^−3^
tissueVol_T1	1.43 × 10^−3^
Volume_cu_mm_Tumor	2.22 × 10^−3^
SER_Partial_tissue_vol_cu_mm_T1	3.77 × 10^−3^
WashinRate_map_inverse_difference_is_homom_tumor	5.84 × 10^−3^
WashinRate_map_Homogeneity1_tumor	5.84 × 10^−3^
WashinRate_map_Homogeneity2_tumor	5.84 × 10^−3^
SER_Washout_tissue_vol_cu_mm_T1	6.95 × 10^−3^
WashinRate_map_inverse_difference_normalized_tumor	6.95 × 10^−3^
WashinRate_map_Dissimilarity_tumor	8.31 × 10^−3^
WashinRate_map_difference_entropy_tumor	8.40 × 10^−3^
Max_Probability_tissue_T1	8.76 × 10^−3^
WashinRate_map_Homogeneity2_tissue_T1	9.56 × 10^−3^
WashinRate_map_inverse_difference_is_homom_tissue_T1	9.96 × 10^−3^
WashinRate_map_Homogeneity1_tissue_T1	9.96 × 10^−3^
Inf_mea_of_corr1_Tumor	9.96 × 10^−3^
WashinRate_map_inverse_difference_moment_normalized_tumor	1.15 × 10^−2^
SER_Total_tissue_vol_cu_mm_T1	1.15 × 10^−2^
WashinRate_map_Max_Probability_tissue_T1	1.37 × 10^−2^
Energy_tissue_T1	1.40 × 10^−2^
WashinRate_map_Energy_tissue_T1	1.40 × 10^−2^
BreastVol	1.40 × 10^−2^
WashinRate_map_Contrast_tumor	1.40 × 10^−2^
WashinRate_map_Dissimilarity_tissue_T1	1.78 × 10^−2^
WashinRate_map_inverse_difference_normalized_tissue_T1	1.78 × 10^−2^
WashinRate_map_Entropy_tissue_T1	1.78 × 10^−2^
Grouping_based_proportion_of_3D_tissue_PostCon_Group_1	1.78 × 10^−2^
WashinRate_map_inverse_difference_moment_normalized_tissue_T1	1.84 × 10^−2^
Correlation1_Tumor	1.84 × 10^−2^
WashinRate_map_Contrast_tissue_T1	1.84 × 10^−2^
Correlation2_Tumor	1.84 × 10^−2^
WashinRate_map_difference_entropy_tissue_T1	1.90 × 10^−2^
Entropy_tissue_T1	1.96 × 10^−2^

## Data Availability

Data are contained within the article and Supplementary Materials.

## Supplementary Materials

The following supporting information can be downloaded at: https://www.mdpi.com/article/10.3390/cancers17172912/s1, Figure S1: The MRI feature S1_SER_Partial_tumor_vol_cu_mm is significantly associated with race (*p* < 0.0008). BAA50 (Black or African American 50 years or younger); W50 (White 50 years or younger); Figure S2: The MRI feature S2_SER_Total_tumor_vol_cu_mm is significantly associated with race (*p* < 0.0008). BAA50 (Black or African American 50 years or younger); W50 (White 50 years or younger); Figure S3: The MRI feature S3_tissueVol_T1 is significantly associated with race (*p* < 0.0008). BAA50 (Black or African American 50 years or younger); W50 (White 50 years or younger); Figure S4: The MRI feature S4_Volume_cu_mm_Tumor is significantly associated with race (*p* < 0.0008). BAA50 (Black or African American 50 years or younger); W50 (White 50 years or younger); Figure S5: The MRI feature S5_SER_Partial_tissue_vol_cu_mm_T1 is significantly associated with race (*p* < 0.0008). BAA50 (Black or African American 50 years or younger); W50 (White 50 years or younger); Figure S6: The MRI feature S6_WashinRate_map_inverse_difference_is_homom_tumor is significantly associated with race (*p* < 0.0008). BAA50 (Black or African American 50 years or younger); W50 (White 50 years or younger); Figure S7: The MRI feature S7_WashinRate_map_Homogeneity1_tumor is significantly associated with race (*p* < 0.0008). BAA50 (Black or African American 50 years or younger); W50 (White 50 years or younger); Figure S8: The MRI feature S8_WashinRate_map_Homogeneity2_tumor is significantly associated with race (*p* < 0.0008). BAA50 (Black or African American 50 years or younger); W50 (White 50 years or younger); Figure S9: The MRI feature S9_SER_Washout_tissue_vol_cu_mm_T1 is significantly associated with race (*p* < 0.0008). BAA50 (Black or African American 50 years or younger); W50 (White 50 years or younger); Figure S10: (**A**). Workflow depiction of the methods used to apply machine learning algorithms to predict disease recurrence and molecular subtype classifications. (**B**). Workflow depiction of the methods used to identify imaging features that had significant associations with race; Table S1: Recurrence Events: Top predictive features differ between white and black patients; Table S2: Groupings of 529 algorithmically derived features in contrast-enhanced MRI. Groupings are based on data type used for their extraction and the type of calculation applied (adapted from Saha et al., 2017 [24]); Table S3: Contingency table of race and grade. Table S4: Additional metrics of machine learning performance on classifying subtype using different data modalities; Table S5: Additional metrics of Machine Learning Performance on predicting recurrence events; Table S6: Additional metrics of Machine Learning Performance on predicting Nottingham Grades.
