# Supplementary material for: Radiomics Combined with Transcriptomics Improves Prediction of Breast Cancer Recurrence, Molecular Subtype and Grade
Source: Cancers (Basel). 2025 Sep 5;17(17):2912. doi: 10.3390/cancers17172912 (PMC12427701; doi:10.3390/cancers17172912)
Supplement: Supplementary file 1 [file cancers-17-02912-s001.zip › Figure_S10.pdf]

**Supplementary Figures S10**

**A**

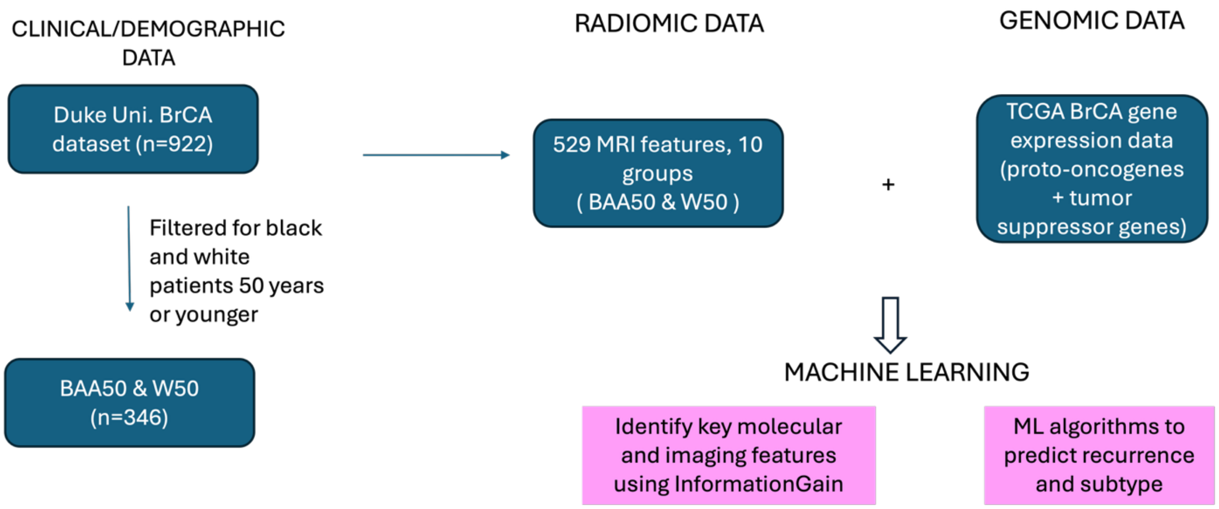


**B**

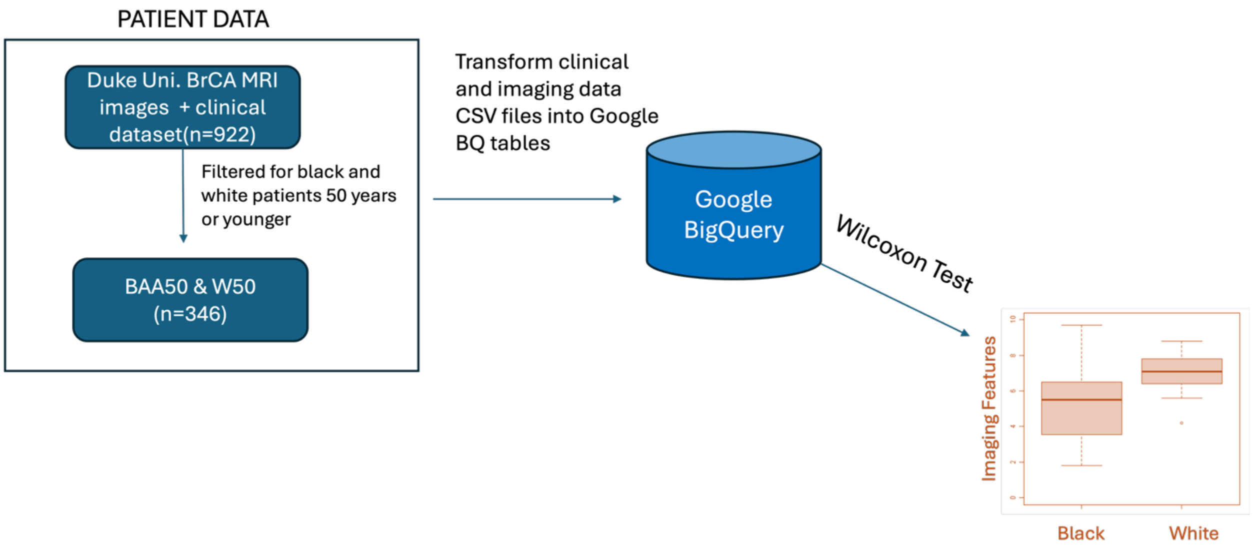


Figure legend:

**A.** Workflow depiction of the methods used to apply machine learning algorithms to predict disease recurrence and molecular subtype classifications. **B.** Workflow depiction of the methods used to identify imaging features that had significant associations with race.
