# Supplementary material for: Radiomics Combined with Transcriptomics Improves Prediction of Breast Cancer Recurrence, Molecular Subtype and Grade
Source: Cancers (Basel). 2025 Sep 5;17(17):2912. doi: 10.3390/cancers17172912 (PMC12427701; doi:10.3390/cancers17172912)
Supplement: Supplementary file 1 [file cancers-17-02912-s001.zip › Table_S1.docx]

**Supplementary Table S1**. Recurrence Events: Top predictive features differ between white and black patients

| **Imaging Feature Categories** | **Black Patients** | **White Patients** |
| --- | --- | --- |
| Breast and FGT Volume Features | BreastVol (p_adj= 0.014) | tissueVol_PostCon (p_adj= 0.001) |
| Tumor Size and Morphology | Median_Euler_No_Tumor (p_adj = 0.988) | Median_Euler_No_Tumor (p_adj= 0.988) |
| FGT Enhancement | Grouping_based_proportion_of_3D_tissue_PostCon_Group_2 (p_adj= 0.258) | Grouping_based_proportion_of_tumor_voxels_3D_tumor_Group_2 (p_adj=1) |
| Tumor Enhancement | WashinRate_map_mean_tumor (p_adj=1) | Grouping_based_proportion_of_tumor_voxels_2D_tumorSlice_Group_2 (p_adj=1) |
| Combining Tumor and FGT Enhancement | F1_DT_POSTCON__T11_0_05_T12_0_1_ (p_adj=0.865) | F1_DT_POSTCON__T11_0_05_T12_0_1_ (p_adj=0.865) |
| FGT Enhancement Texture | WashinRate_map_Cluster_Prominence_tissue_T1 (p_adj=0.209) | SER_map_Correlation2_tissue_PostCon (p_adj=0.635) |
| Tumor Enhancement Texture | _2nd_DFT_CoeffMap_Momment_Invariant_4_3D_tumor (p_adj=0.321) | WashinRate_map_Entropy_tumor (p_adj=1) |
| Tumor Enhancement Spatial Heterogeneity | EnhancementClusterDiscontinuity_Tumor (p_adj=0.608) | globalMoransISERMap_Tumor (p_adj= 0.785) |
| FGT Enhancement Variation | WashinRate_map_kurtosis_tissue_T1 (p_adj=0.368) | Grouping_based_variance_of_peak_enhancement_slope_3D_tissue_PostCon_Group_2 (p_adj=0.754) |
| Tumor Enhancement Variation | WashinRate_map_std_dev_tumor (p_adj=1) | WashinRate_map_kurtosis_tumor (p_adj=0.258) |
