# Supplementary figures and images for: Radiomics Combined with Transcriptomics Improves Prediction of Breast Cancer Recurrence, Molecular Subtype and Grade
Source: Cancers (Basel). 2025 Sep 5;17(17):2912. doi: 10.3390/cancers17172912 (PMC12427701; doi:10.3390/cancers17172912)

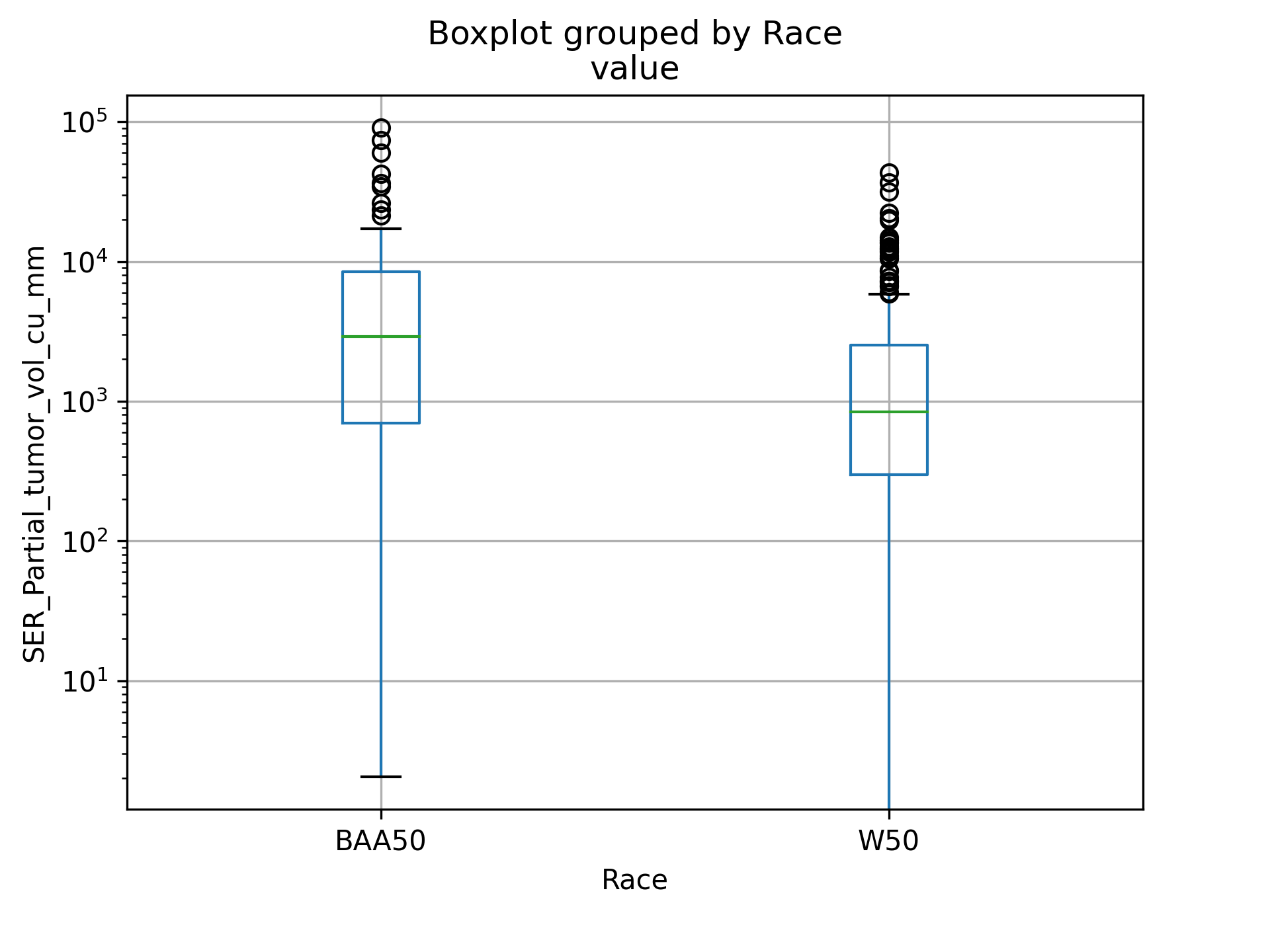

Supplement: Supplementary file 1 [file cancers-17-02912-s001.zip › Figure_S1_SER_Partial_tumor_vol_cu_mm.png]

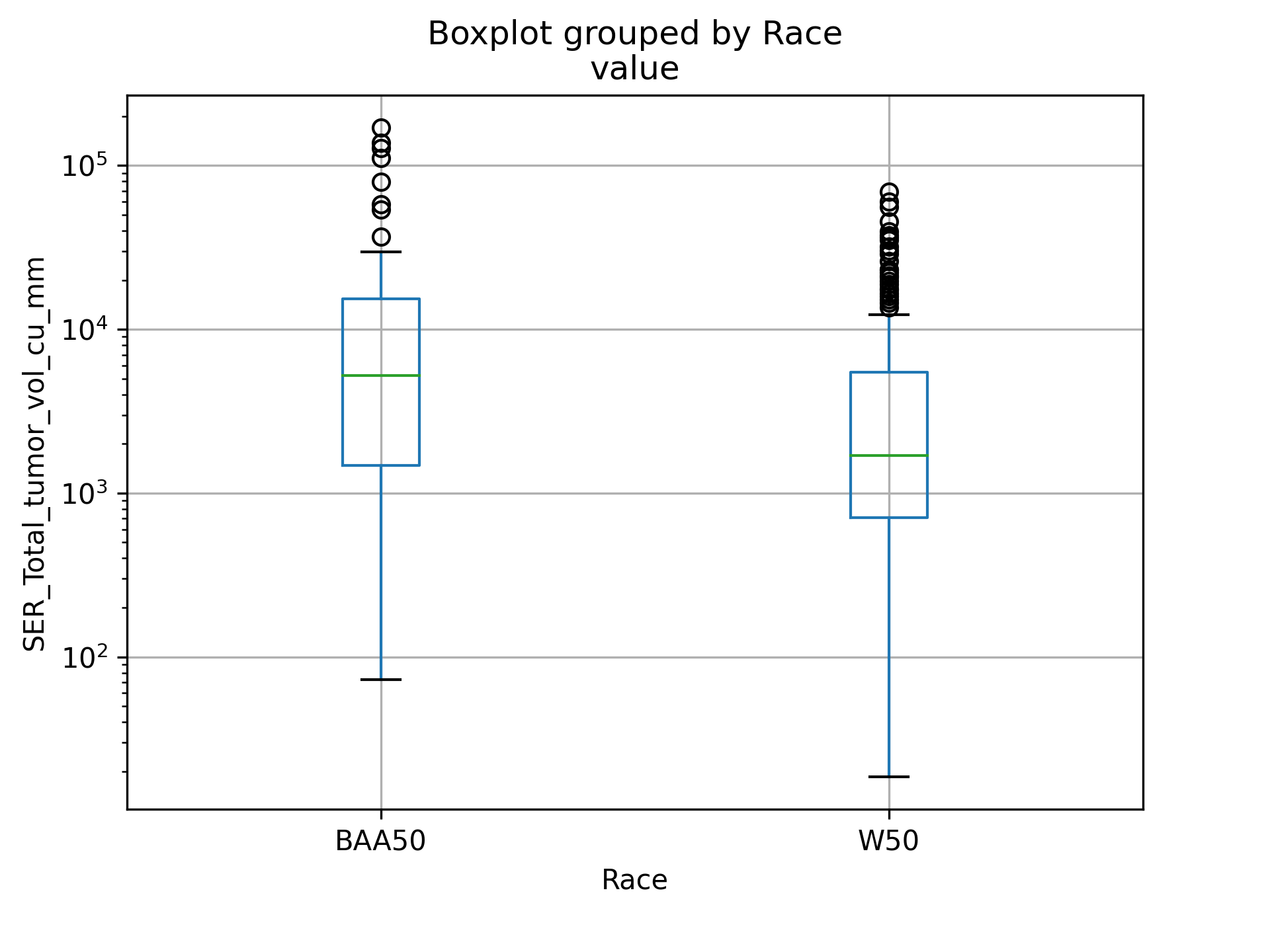

Supplement: Supplementary file 1 [file cancers-17-02912-s001.zip › Figure_S2_SER_Total_tumor_vol_cu_mm.png]

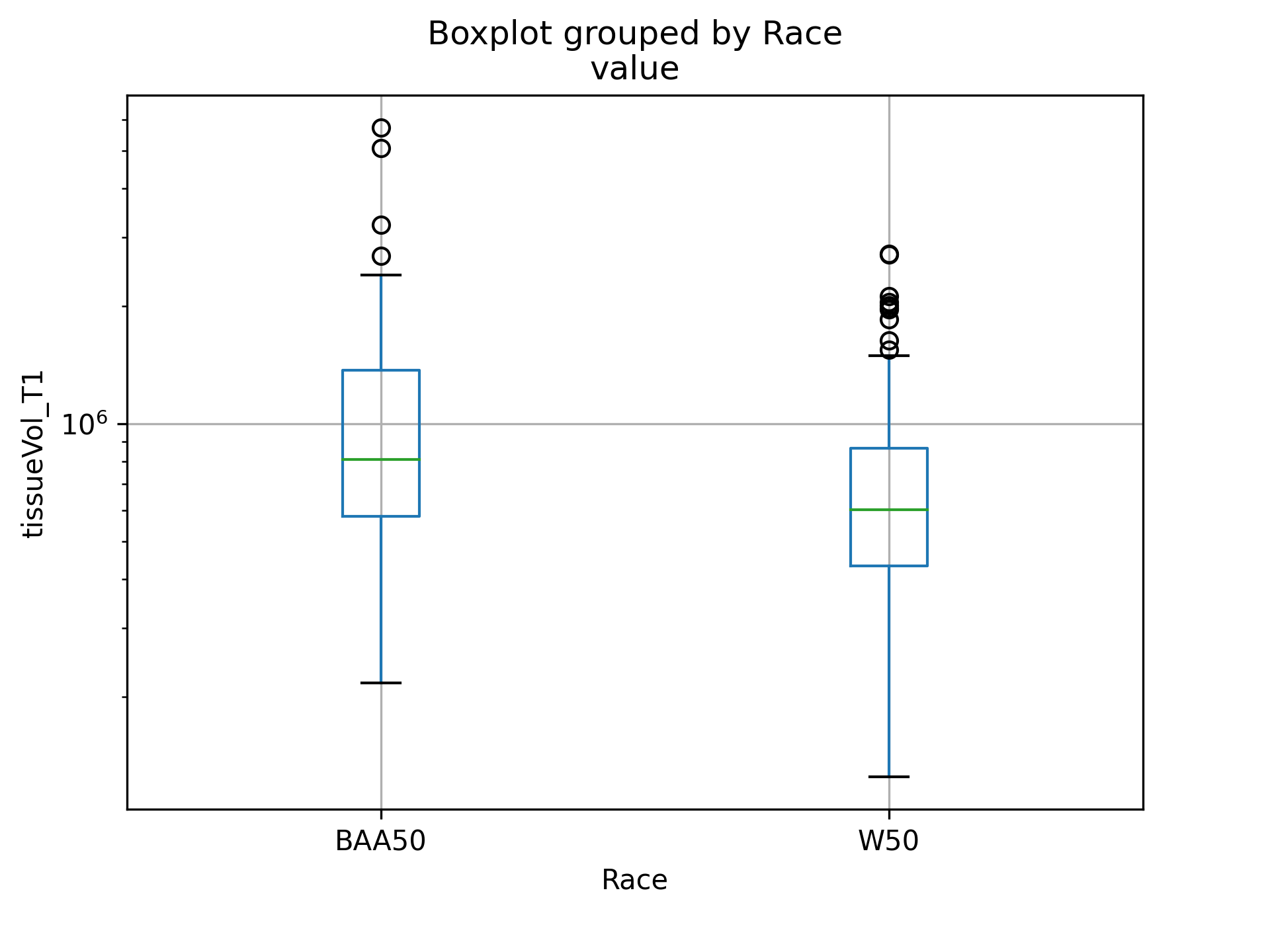

Supplement: Supplementary file 1 [file cancers-17-02912-s001.zip › Figure_S3_tissueVol_T1.png]

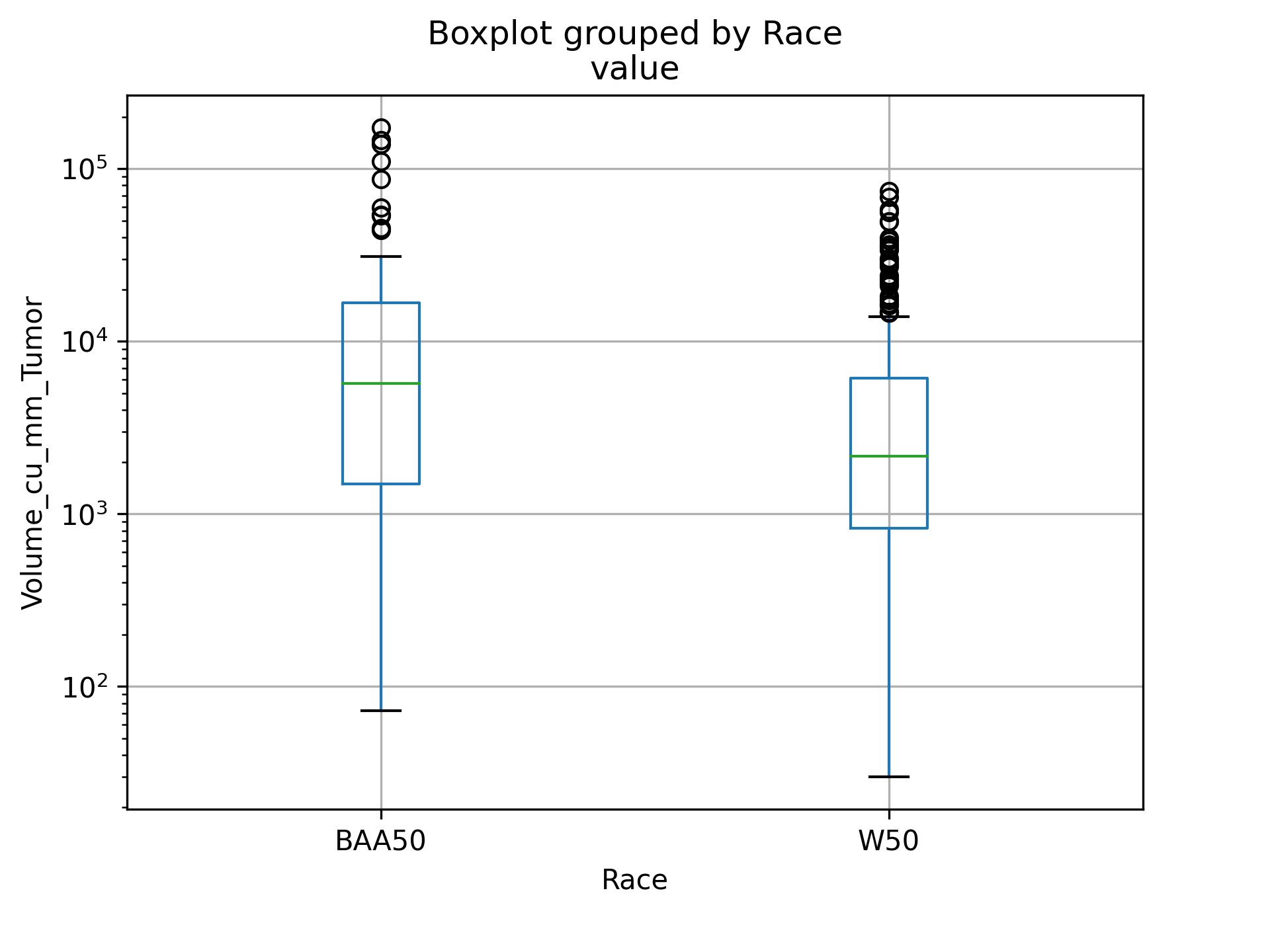

Supplement: Supplementary file 1 [file cancers-17-02912-s001.zip › Figure_S4_Volume_cu_mm_Tumor.png]

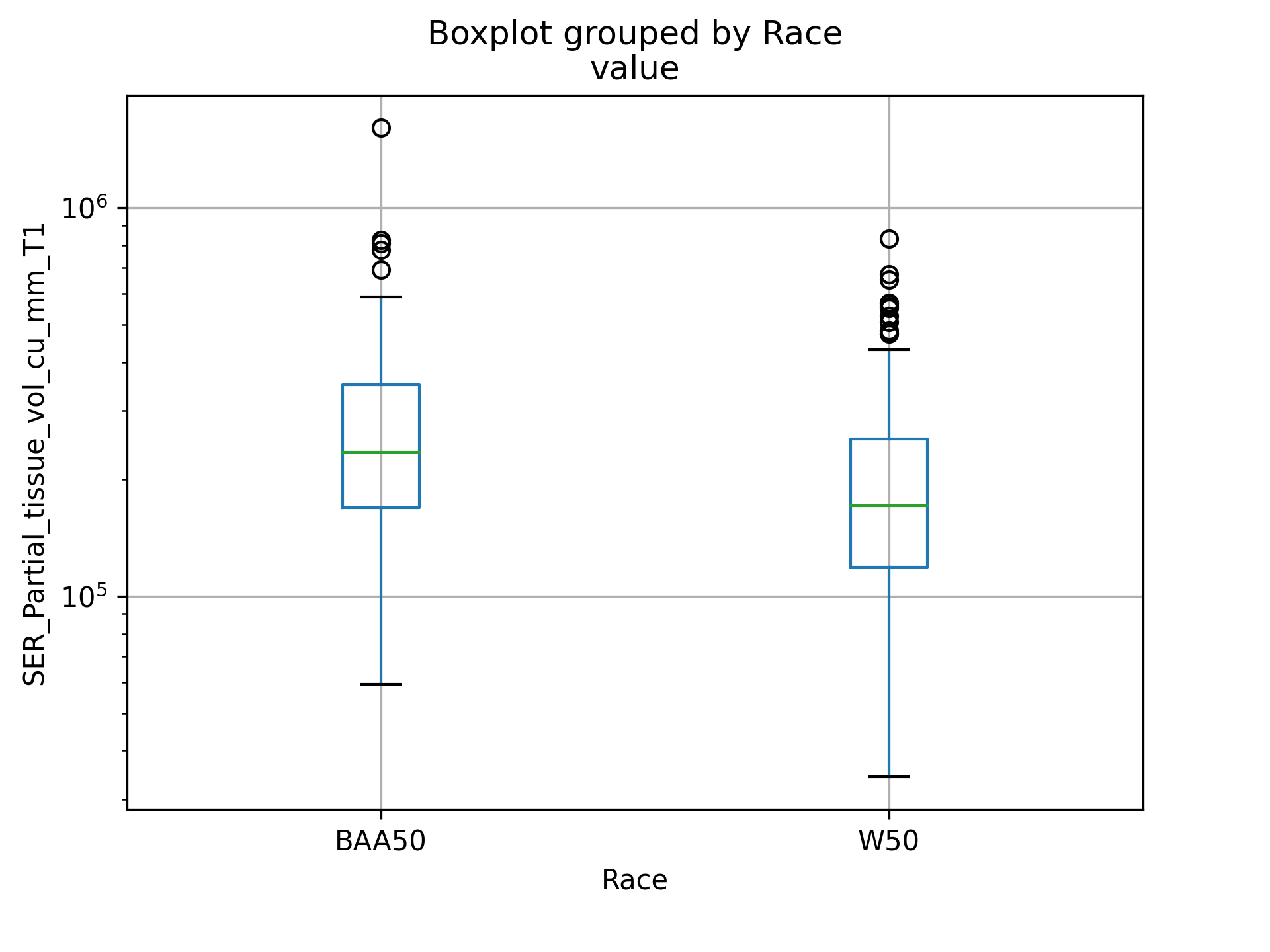

Supplement: Supplementary file 1 [file cancers-17-02912-s001.zip › Figure_S5_SER_Partial_tissue_vol_cu_mm_T1.png]

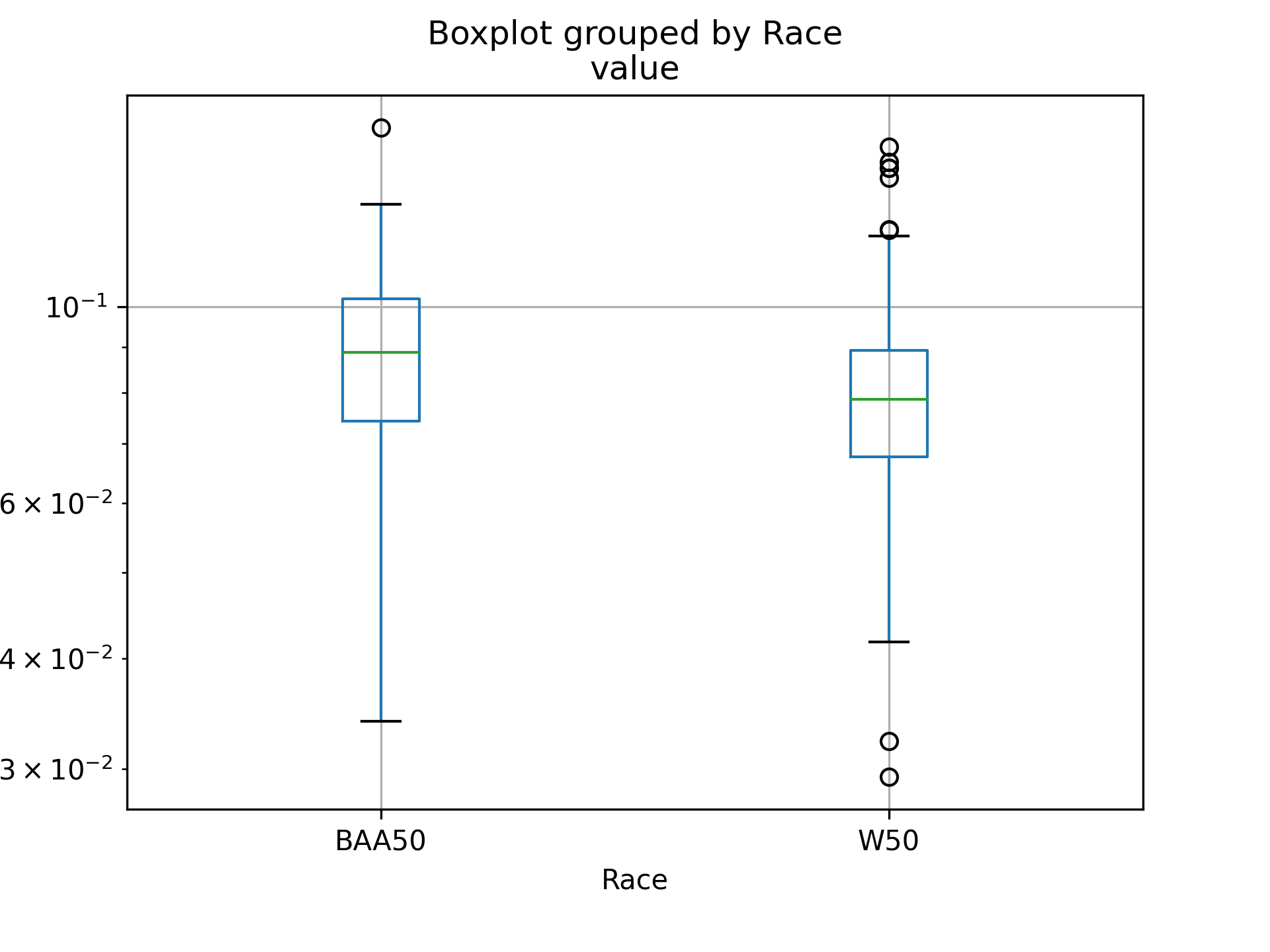

Supplement: Supplementary file 1 [file cancers-17-02912-s001.zip › Figure_S6_WashinRate_map_inverse_difference_is_homom_tumor.png]

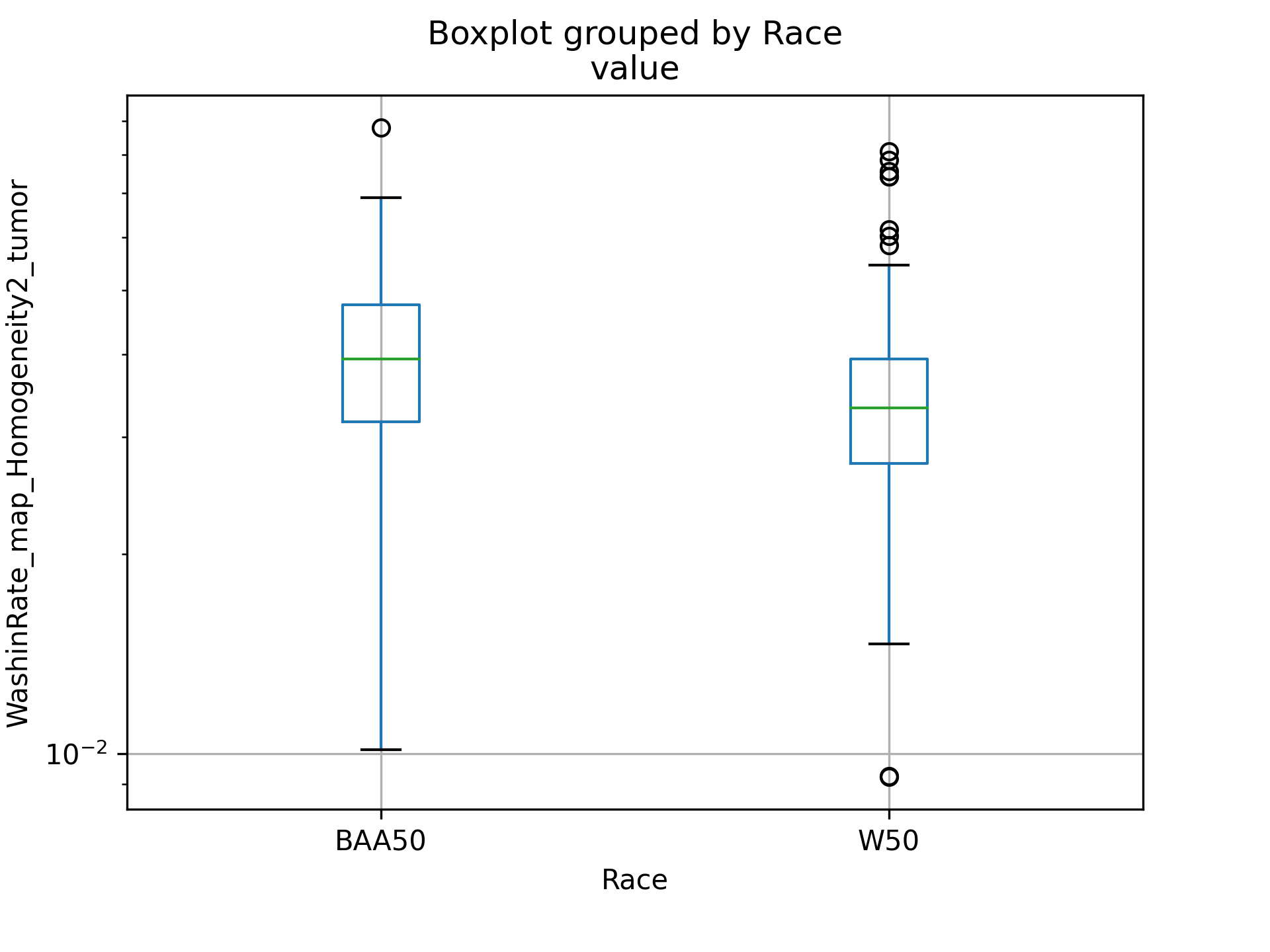

Supplement: Supplementary file 1 [file cancers-17-02912-s001.zip › Figure_S8_WashinRate_map_Homogeneity2_tumor.png]

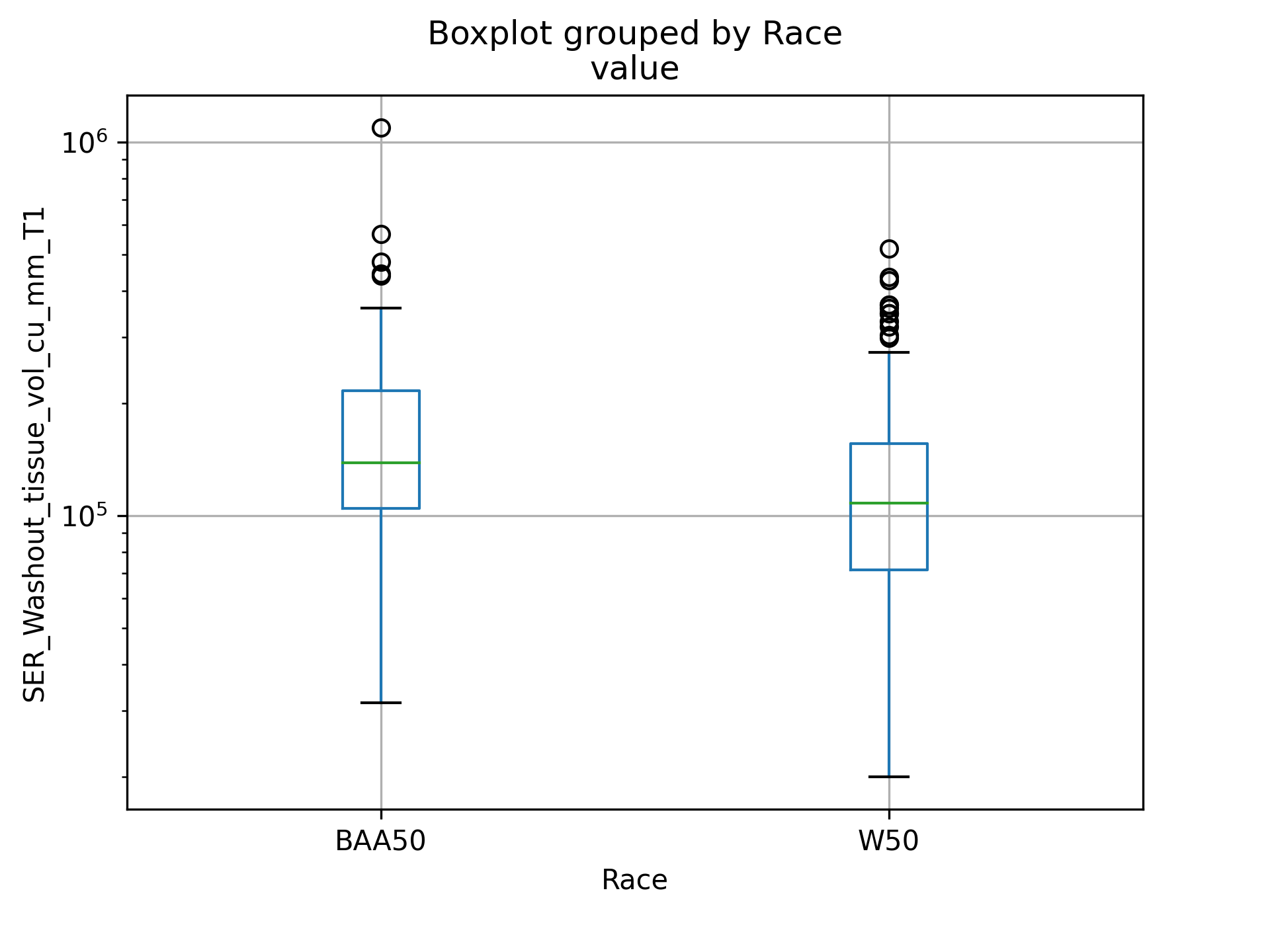

Supplement: Supplementary file 1 [file cancers-17-02912-s001.zip › Figure_S9_SER_Washout_tissue_vol_cu_mm_T1.png]
